# Farrerol Relieve Lipopolysaccharide (LPS)-Induced Mastitis by Inhibiting AKT/NF-κB p65, ERK1/2 and P38 Signaling Pathway

**DOI:** 10.3390/ijms19061770

**Published:** 2018-06-14

**Authors:** Yanwei Li, Qian Gong, Wenjin Guo, Xingchi Kan, Dianwen Xu, He Ma, Shoupeng Fu, Juxiong Liu

**Affiliations:** College of Veterinary Medicine, Jilin University, Changchun 130062, China; ywli17@mails.jlu.edu.cn (Y.L.); qianqiangong2018@163.com (Q.G.); guowj17@mails.jlu.edu.cn (W.G.); kanxc16@mails.jlu.edu.cn (X.K.); xudw9913@mails.jlu.edu.cn (D.X.); mahe9916@mails.jlu.edu.cn (H.M.)

**Keywords:** mastitis, farrerol, LPS, NF-κB, Mitogen-activated protein kinase (MAPK)

## Abstract

Farrerol has been proved to have an anti-inflammatory effect. However, the effects of farrerol on mastitis have not been investigated. This study was aimed to investigate the effect and mechanism of farrerol in lipopolysaccharide (LPS)-induced mouse mastitis and LPS-induced inflammatory response of mouse mammary epithelial cells (mMECs). In vivo, LPS were injected to the tetrad pair of nipples for establishing mouse mastitis, and then tested the effect of farrerol on histopathological changes, inflammatory response and activation degree of protein kinase B (AKT), nuclear factor-kappa B p65 (NF-κB p65), p38, extracellular regulated protein kinase (ERK1/2). In vitro, the mMECs were incubated by farrerol for 1 h following by stimulating with LPS, and then the inflammatory response and the related signaling pathways were detected. The in vivo results found that farrerol could improve pathological injury of mammary gland, attenuate the activity of myeloperoxidase (MPO), inhibit the production of pro-inflammatory mediators and the phosphorylation of AKT, NF-κB p65, p38 and ERK1/2. The in vitro results also found farrerol inhibited inflammatory response and the related signaling pathways. Collectively, this study revealed that farrerol inhibits the further development of LPS-induced mastitis by inhibiting inflammatory response via down regulating phosphorylation of AKT, NF-κB p65, p38, and ERK1/2. These findings suggest that farrerol may be used as an anti-inflammatory drug for mastitis.

## 1. Introduction

Bovine mastitis is an important disease that affects dairy industry worldwide. It can cause a decline of milk production and quality, thereby causing great economic loss to the dairy industry [1]. Bovine mastitis is caused by a variety of factors, and the most common factor is the infection of pathogens [2,3]. The characteristics of mastitis mainly manifest as mammary swelling, inflammatory cell infiltration, and damage of the mammary bubble [4,5,6]. Mastitis is caused by a variety of microorganisms including Gram-negative bacteria [7], and lipopolysaccharide (LPS) is a pathogen-associated molecular pattern from cell well of Gram-negative bacteria. Mammary epithelial cells are involved in the first line of defense against the invasion of the mammary gland and playing an important role in the early stage of the infection [8]. LPS can bind to toll-like receptor 4 (pattern-recognition receptor) of mammary epithelial cells, and activate multiple signaling pathways nuclear factor-kappa B (NF-κB), mitogen-activated protein kinase (MAPK) and other signaling pathways [9]. The activation of these signal pathways will lead to the production of various pro-inflammatory mediators (tumor necrosis factor-α (TNF-α), interleukin-1 beta (IL-1β), interleukin 6 (IL-6), induced nitric oxide synthase (iNOS) and cyclooxygense-2 (COX-2)), which are capable of amplifying the process of inflammation [10]. So far, the main method of bovine mastitis treatment is to take antibiotics. At present, many studies have shown that the addition of anti-inflammatory drugs to antimicrobial treatment of bovine mastitis obtained more economic benefits [11,12]. Therefore, in addition to antibacterial, anti-inflammatory in the treatment of mastitis can not be ignored.

**Figure 1:**
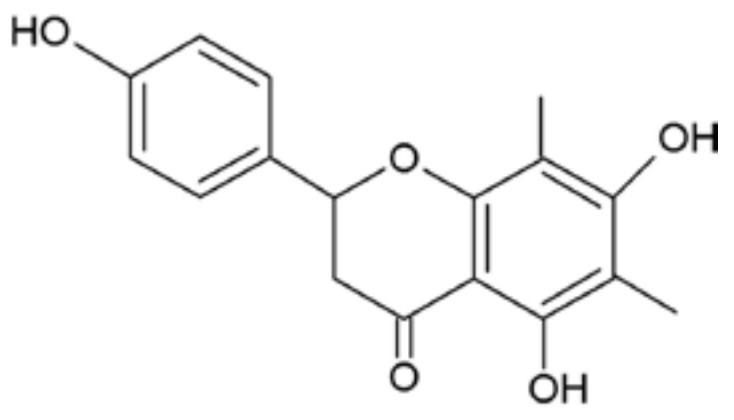
Chemical structure of farrerol.

Farrerol (Figure 1), a dihydroflavone compound extracted from leaves of *Rhododendron dauricum* L. has the pharmacological action of relieving cough and expectoration [13]. Modern pharmacological studies have shown that farrerol has antibacterial, anti-inflammatory, anti-cancer, immunosuppressive effects and it can also inhibit vascular smooth muscle proliferation [13,14]. Some Chinese herbs have been reported to have a good effect on the treatment of mastitis; however, there is no report about the study of farrerol on mastitis [15,16]. Therefore, this study mainly investigated the effect and mechanism of farrerol in LPS-induced mouse mastitis and LPS-induced inflammatory response of mouse mammary epithelial cells (mMECs).

## 2. Results

### 2.1. Effect of Farrerol on Histopathological Changes

**Figure 2:**
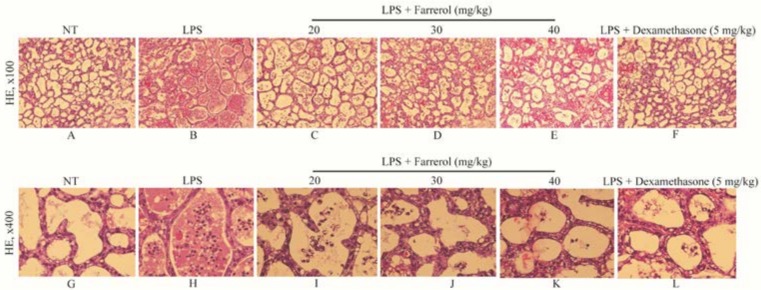
Effects of farrerol on the pathological changes of mammary gland ((**A**–**F**), ×100 and (**G**–**L**), ×400). Mammary glands were collected after lipopolysaccharide (LPS) injection 24 h. The fixed tissue blocks were fixed, dehydrated, transparent, dipped wax, embedded, sliced, made of the paraffin sections and then stained by means of hematoxylin–eosin (HE) staining. Representative histopathological changes of mammary tissues from each group: no treatment (NT) group (**A**,**G**); LPS group (**B**,**H**); LPS + farrerol (20 mg/kg) group (**C**,**I**); LPS + farrerol (30 mg/kg) group (**D**,**J**); LPS + farrerol (40 mg/kg) group (**E**,**K**); LPS + dexamethasone (5 mg/kg) group (**F**,**L**). Histomorphology and pathology showed that treatment with farrerol can alleviated LPS-induced pathological changes.

The histopathological images of all groups (no treatment (NT), LPS, LPS + farrerol (20, 30, 40 mg/kg), LPS + dexamethasone (5 mg/kg)) were shown in Figure 2. The NT group displayed no abnormal histopathological changes occurred (Figure 2A,G), while, in the LPS group, the mammary gland acini were hyperemia oedema and infiltrated with a large number of neutrophils (Figure 2B,H). In contrast, the mammary gland histopathological changes of the LPS + farrerol (20, 30, 40 mg/kg) groups were improved and the number of neutrophils in mammary glands were reduced (Figure 2C–E,I–K).

### 2.2. Effect of Farrerol on Myeloperoxidase (MPO) Activity

**Figure 3:**
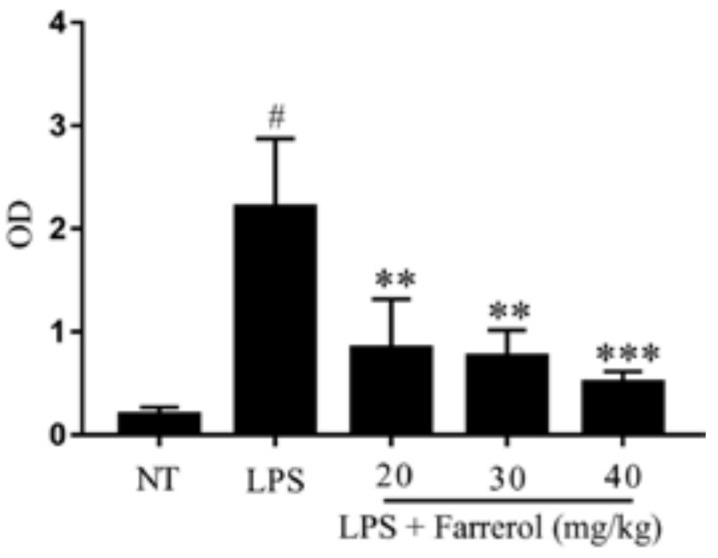
Effects of farrerol on the myeloperoxidase (MPO) activity in mammary gland. The values were presented as the means ± SEM of three independent experiments (*n* = 3). # *p* < 0.05 vs. NT group; ** *p* < 0.01 and *** *p* < 0.001 vs. LPS group.

The activity of MPO was a hallmark of inflammatory cell infiltration. In order to get quantitative date of inflammatory, we examined MPO activity in mammary gland. The result showed that MPO activity was significantly increased in LPS group (*p* < 0.05). However, compared with the LPS group, the MPO activity were dose-dependently decreased in LPS + farrerol (20, 30, 40 mg/kg) groups (Figure 3).

### 2.3. Effect of Farrerol on the Production of Pro-Inflammatory Mediators in Mammary Gland

**Figure 4:**
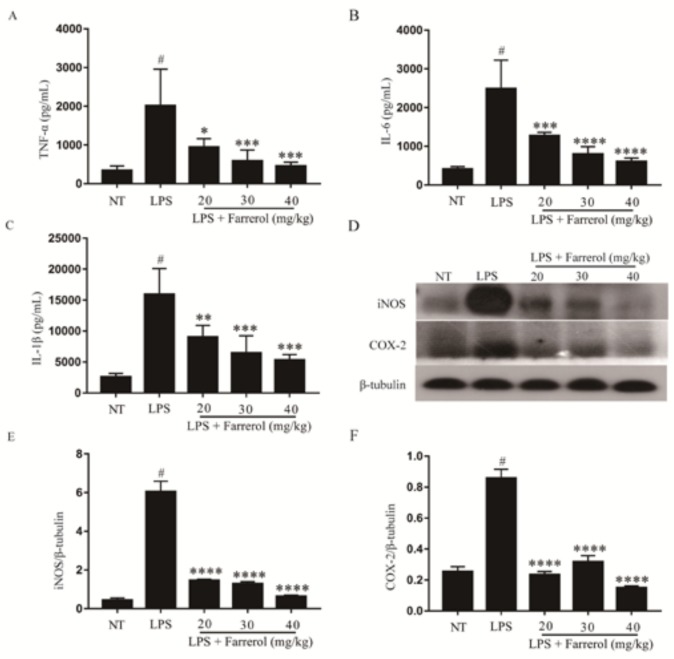
The levels of TNF-α (**A**); IL-6 (**B**) and IL-1β (**C**) in the homogenate of mouse mammary glands including NT group, LPS group and LPS + farrerol (20, 30, 40 mg/kg) group; The protein levels of iNOS (**D**,**E**) and COX-2 (**D**,**F**) were measured by Western blot. Data are presented as mean ± SEM (*n* = 3). # *p* < 0.05 vs. NT group; * *p* < 0.05, ** *p* < 0.01, *** *p* < 0.001 and **** *p* < 0.0001 vs. LPS group.

In the process of inflammation, pro-inflammatory mediators, which include TNF-α, IL-6, IL-1β, iNOS and COX-2, would be produced in sites of inflammation. The effect of farrerol on the protein levels of TNF-α, IL-6, IL-1β, iNOS and COX-2 were detected by enzyme-linked immunosorbent assay (ELISA) or Western blot. The results showed that the pro-inflammatory mediators in LPS group were significantly increased than NT group (*p* < 0.05). However, compared to the LPS group, the protein levels of pro-inflammatory mediators were significantly decreased in a dose-dependent manner in the LPS + farrerol (20, 30, 40 mg/kg) group (*p* < 0.05). These results indicated that farrerol could inhibit the protein levels of TNF-α, IL-6, IL-1β, iNOS and COX-2 in mammary glands from LPS-induced mouse mastitis model (Figure 4).

### 2.4. Effect of Farrerol on the Activity of AKT and NF-κB Signaling Pathways in LPS-Induced Mouse Mastitis

**Figure 5:**
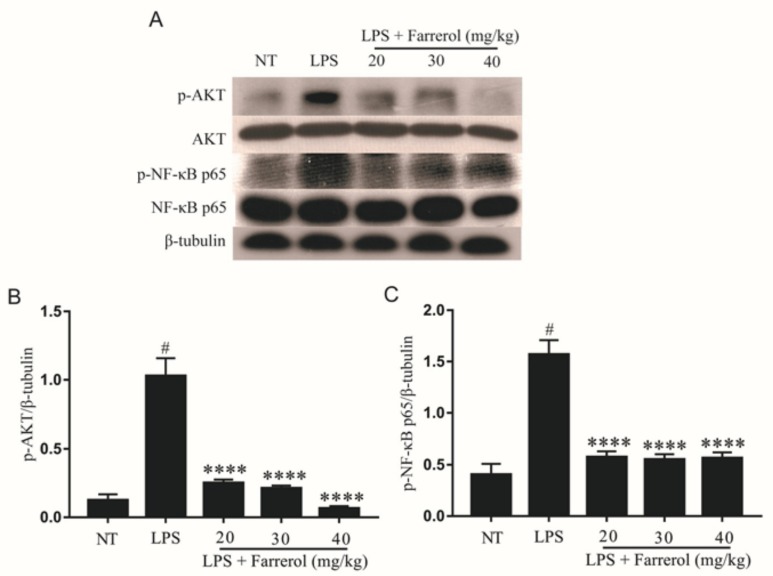
Farrerol inhibited the phosphorylation of protein kinase B (AKT) and NF-κB signal pathways protein in LPS-induced mouse mastitis. The protein levels of p-AKT (**A**,**B**) and p-NF-κB p65 (**A**,**C**) were measured by Western blot. Data were presented as mean ± SEM (*n* = 3). # *p* < 0.05 vs. NT group; **** *p* < 0.0001 vs. LPS group.

The AKT and NF-κB signal pathways are concerned with the inflammatory response, and their phosphorylation levels increased in mastitis. To elucidate the mechanism of farrerol anti-neuroinflammation, we examined the effect of farrerol on phosphorylation of AKT and NF-κB p65. The Western blot results showed that the phosphorylation of AKT and NF-κB p65 in the LPS group were significantly increased compared with the NT group (*p* < 0.05), and farrerol could dose-dependently inhibit the phosphorylation of AKT and NF-κB p65 compared with the LPS group (Figure 5).

### 2.5. Effect of Farrerol on the Activity of MAPK Signaling Pathways in LPS-Induced Mouse Mastitis

**Figure 6:**
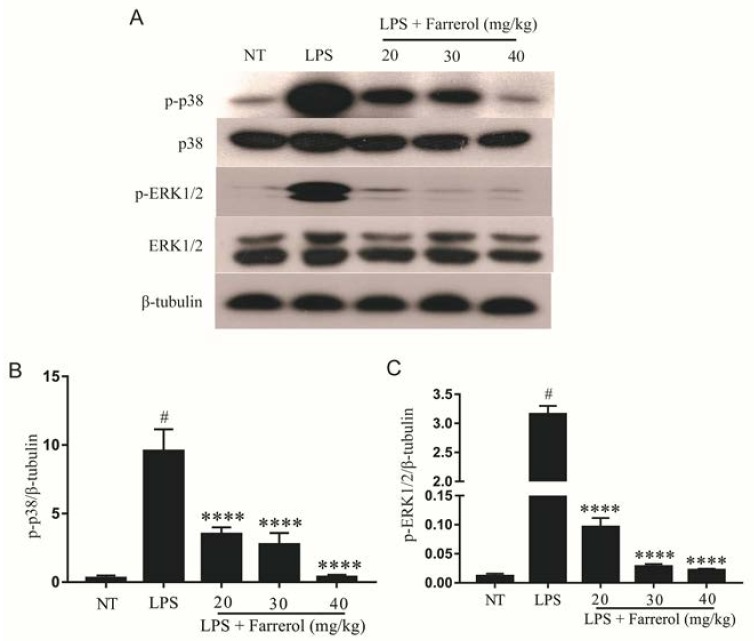
Farrerol inhibited the phosphorylation of ERK1/2, p38 in LPS-induced mouse mastitis. The phosphorylation of p38 (**A**,**B**) and ERK1/2 (**A**,**C**) were measured by Western blot. Data are presented as mean ± SEM (*n* = 3). # *p* < 0.05 vs. NT group; **** *p* < 0.0,001 vs. LPS group.

MAPK signaling pathways are also concerned with inflammatory response, so we measured the phosphorylation of p38 and ERK1/2 proteins. The Western blot results indicated that the phosphorylation of p38 and ERK1/2 in the LPS group were significantly increased compared with the NT group (*p* < 0.05), and farrerol could dose-dependently inhibit the phosphorylation of p38 and ERK1/2 compared with the LPS group (Figure 6).

### 2.6. Effect of Farrerol on mMECs Viability

**Figure 7:**
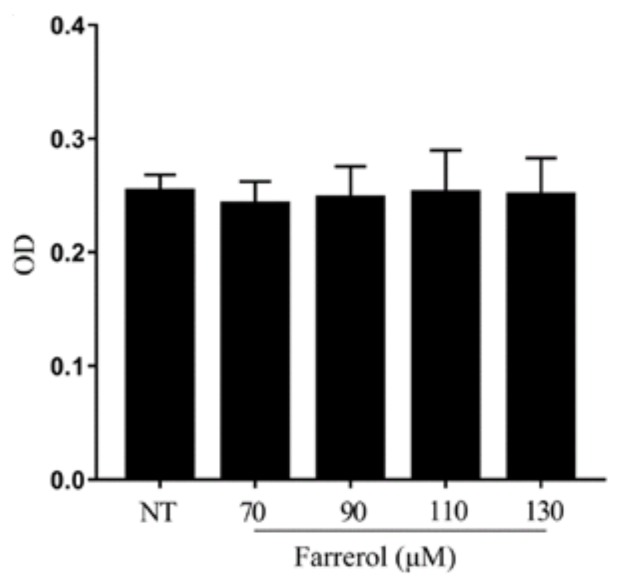
Effects of farrerol on the cell viability of mouse mammary epithelial cells (mMECs) cultured with different concentrations of farrerol (70, 90, 110 and 130 μM). MMECs viability were determined by CCK-8 assay. Data are presented as mean ± SEM (*n* = 6).

The potential cytotoxicity of farrerol on mMECs was analyzed by cell counting kit-8 (CCK-8) assay. As shown in Figure 7, the viability of the mMECs was not affected by farrerol (70, 90, 110, 130 μM), so we have chosen the concentration of 90, 110, 130 μM.

### 2.7. Effect of Farrerol on Inflammatory Response in LPS-Stimulated mMECs

**Figure 8:**
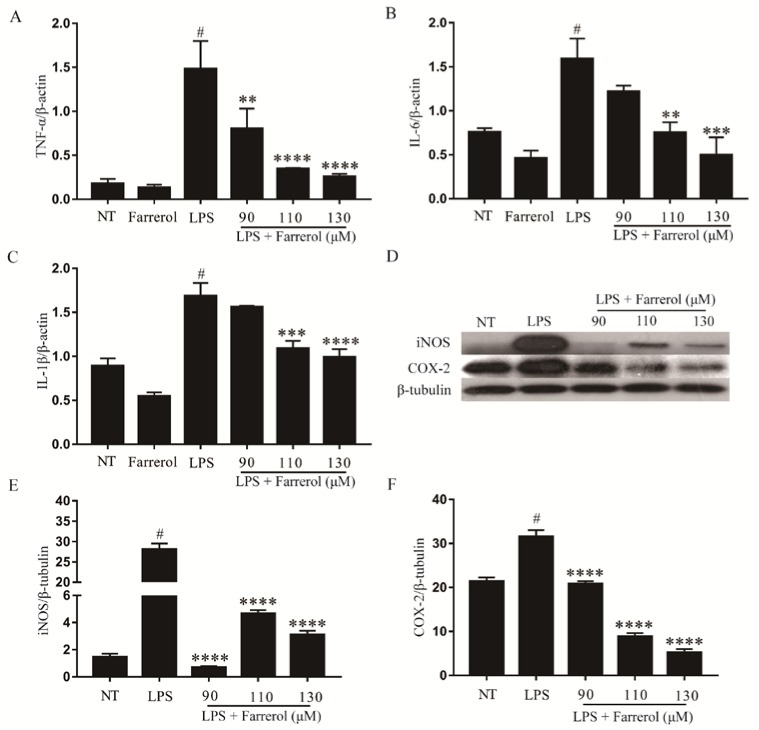
The effect of farrerol on pro-inflammatory mediator’s production in LPS-stimulated mMECs. MMECs were divided into six groups: NT group, Farrerol group, LPS group, LPS + farrerol (90, 110, 130 μM) group. The mRNA levels of TNF-α (**A**); IL-6 (**B**) and IL-1β (**C**) were measured by real-time PCR. The protein levels of iNOS (**D**,**E**) and COX-2 (**D**,**F**) were measured by Western blot. Data are presented as mean ± SEM (*n* = 3). # *p* < 0.05 vs. NT group; ** *p* < 0.01, *** *p* < 0.001 and **** *p* < 0.0001 vs. LPS group.

The mRNA levels of TNF-α, IL-6 and IL-1β in LPS-stimulated mMECs were determined by real-time PCR, and the protein levels of iNOS and COX-2 were examined by Western blot. The results are shown in Figure 8, the mRNA levels of TNF-α (A), IL-6 (B) and IL-1β (C) were significantly increased after stimulated with LPS (*p* < 0.05), and farrerol inhibited this effect in a dose-dependent manner (Figure 8). The expression of iNOS (D,E) and COX-2 (D,F) were significantly increased in the LPS group, and significantly decreased in the LPS + farrerol (90, 110, 130 μM) groups (*p* < 0.05).

### 2.8. Effect of Farrerol on the Activity of NF-κB, AKT, and MAPK Signaling Pathways in LPS-Stimulated mMECs

**Figure 9:**
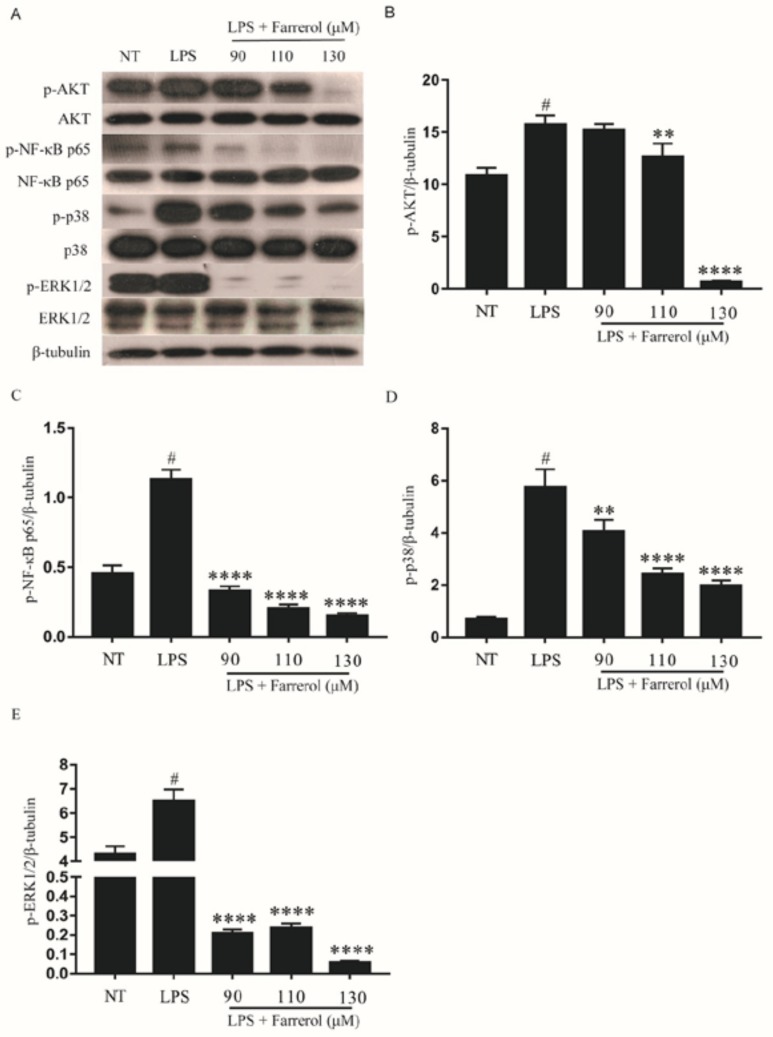
Farrerol inhibited the phosphorylation of AKT, NF-κB, p38 and ERK1/2 in LPS-stimulated mMECs. The phosphorylation AKT (**A**,**B**); NF-κB p65 (**A**,**C**); p38 (**A**,**D**); and ERK1/2 (**A**,**E**) were measured by Western blot. Data were presented as mean ± SEM (*n* = 3). # *p* < 0.05 vs. NT group; ** *p* < 0.01 and **** *p* < 0.0,001 vs. LPS group.

The phosphorylation of AKT, NF-κB, p38 and ERK1/2 in LPS-stimulated mMECs was examined by Western blot. As shown in Figure 9, the phosphorylation of AKT, NF-κB p65, p38 and ERK1/2 were significantly increased in the LPS group(*p* < 0.05), and farrerol could inhibit this effect in a dose-dependent manner (*p* < 0.05).

## 3. Discussion

Studies have shown that farrerol has a therapeutic effect on ovalbumin (OVA)-Induced Allergic Asthma in mice [17]. The effects of farrerol on mastitis have not been reported in any study. This study found that farrerol could improve pathological injury of mammary glands, attenuate the activity of MPO, inhibit the production of pro-inflammatory mediators (TNF-α, IL-6, IL-1β, iNOS and COX-2) and the phosphorylation of AKT, NF-κB p65, p38 and ERK1/2 in LPS-induced mouse mastitis. The in vitro results also found farrerol inhibited inflammatory response and phosphorylation of AKT, NF-κB p65, p38 and ERK1/2.

Inflammatory factors play an important role in the development of inflammation [18]. TNF-α is an major inflammatory factor in the early stages of infection and has a chemotactic effect on neutrophils [19]. Like TNF-α, IL-6 and IL-1β play an important role in the process of inflammation. It has been reported that LPS is a major component of Gram-negative bacteria [20]. Studies have shown that LPS-induced inflammatory response in the mammary gland by binding to the toll-like receptor 4 of the mammary epithelial cell membrane [21], which is similar to the inflammatory response of bovine mastitis. Therefore, an LPS-induced mastitis animal model and LPS-induced mammary epithelial cells’ inflammatory response model represent powerful tools for mechanic studies and the identification of potential therapeutic agents. Studies have shown that some plant (like cepharanthine and kaempferol) could attenuate LPS-induced mouse mastitis by suppressing the production of TNF-α, IL-6 and IL-1β [16,22]. In our study, we found in vivo the protein levels of TNF-α, IL-6 and IL-1β were significantly increased in LPS group, and farrerol could significantly inhibit the protein levels of TNF-α, IL-6, and IL-1β in a dose-dependent manner. In LPS-stimulated mMECs, we also found farrerol have this function. Wang et al. have found that farrerol could inhibit the production of IL-6 in LPS-stimulated human gingival fibroblasts [23]. This provides evidence for our results. Studies have shown that iNOS and COX-2 were abundantly expressed in LPS-induced microglial activation [24]. Inhibition of the activity of iNOS and COX-2 could suppress the inflammatory response mediated by microglia and reduce neurodegeneration of neurons in inflammation-induced neurodegenerative animal models [25,26]. Leonurine and emodin can alleviate mouse mastitis by inhibiting the production of iNOS and COX-2 in LPS-induced mastitis model [20,27]. Zhang et al. have proved that farrerol could inhibit inflammatory response via suppress the production of iNOS and COX-2 in IL-1β-stimulated human osteoarthritis chondrocytes [28]. In this study, we also found farrerol inhibited LPS-induced production of iNOS and COX-2 in vivo and in vitro. These results indicate that farrerol inhibits the development of mastitis by inhibiting the production of pro-inflammatory mediators TNF-α, IL-6, IL-1β, iNOS and COX-2.

There are many signal pathways involved in inflammatory response, and some studies have shown that LPS could induce phosphorylation of AKT and subsequently activate the NF-κB signaling pathway [26], and then act as a transcriptional factor regulating the production of pro-inflammatory mediators [29]. In normal conditions, NF-κB is in the cytoplasm, and LPS could induce the phosphorylation of NF-κB p65. In addition, the phosphorylated NF-κB p-p65 could transfer to the nucleus to regulate the transcription of pro-inflammatory mediators [30]. Studies have shown that emodin, oxymatrine and other Chinese traditional medicine monomers could inhibit activation of NF-κB signaling pathway in an LPS-induced mouse mastitis model [31,32,33]. Therefore, in order to further study the anti-inflammatory mechanism of farrerol in mastitis, we detected the phosphorylation levels of AKT and NF-κB p65. The results have shown that farrerol could inhibit the phosphorylation of AKT and NF-κB p65, and suggested farrerol might control the development of inflammation by inhibiting phosphorylation of AKT and NF-κB p65. Studies have shown that farrerol could inhibit the AKT/NF-κB signaling pathway in LPS-stimulated human gingival fibroblasts and IL-1β-stimulated human osteoarthritis chondrocytes [23,28], and this is consistent with our results. Moreover, MAPK signaling pathway plays an important role in the inflammatory response [13]. MAPK signaling pathway regulates the transcription and translation of various pro-inflammatory mediators, and is considered as a potential target for anti-inflammatory therapy [14]. In order to study whether the anti-inflammatory role of farrerol is related to MAPK signaling pathway, we examined the effect of farrerol on the phosphorylation of p38 and ERK1/2. In vivo, the phosphorylation of p38 and ERK1/2 were increased in LPS group. In addition, in LPS + farrerol groups, the phosphorylation of p38 and ERK1/2 were decreased. In vitro, farrerol can also inhibit the phosphorylation of p38 and ERK1/2.These results indicated that farrerol could relieve mastitis by inhibiting the phosphorylation of AKT/NF-κB p65, ERK1/2 and P38.

Collectively, this study revealed that farrerol inhibits the further development of LPS-induced mastitis by inhibiting the production of TNF-α, IL-6, IL-1β, iNOS, and COX-2 via down regulating phosphorylation of AKT, NF-κB p65, ERK1/2, p38.These findings suggest that farrerol may be used as an anti-inflammatory drug for mastitis. In subsequent clinical applications, we will prepare rumen-protected capsules containing farrerol.

## 4. Materials and Methods

### 4.1. Animals

Thirty-six female and eighteen male BALB/c mice were purchased from the Experimental Animal Center of Baiqiuen Medical College of Jilin University (Jilin, China). Two female mice and one male mouse were placed in a separate small cage. All of the mice were provided with enough water and food, and all animal care and experimental procedures in this study were conducted in accordance with the guidelines established by the Jilin University Institutional Animal Care and Use Committee (approved on 27 February 2015, Protocol No. 2015047).

### 4.2. Experimental Model and Grouping Design

Farrerol is a racemic mixture purchased from Shanghai Yuan Ye Bio-technology Co. Ltd. (Shanghai, China). The purity of farrerol reaches more than 99%. Five to seven days after delivery, lactating mice were randomly divided into six groups: a no treatment (NT) group (*n* = 6), LPS group (*n* = 6), LPS + farrerol (20, 30, 40 mg/kg) group (*n* = 6), and LPS + dexamethasone (5 mg/kg) group (*n* = 6). The experimental mice were separated from their pups when injecting farrerol or dexamethasone. The LPS + farrerol (20, 30, 40 mg/kg) groups were treated by intraperitoneal injection with various concentrations of farrerol, and the LPS + dexamethasone (5 mg/kg) groups were treated by intraperitoneal injection with 100 μL dexamethasone (5 mg/kg). After 1 h, all mice of the LPS, LPS + farrerol and LPS + dexamethasone group were anesthetized, followed by these mice’s fourth pair of nipples being injected with 50 μL LPS (0.2 mg/kg) (Sigma-Aldrich, St. Louis, MO, USA). 12 h after LPS injection, the LPS + farrerol and LPS +dexamethasone groups were injected with the same dose of the farrerol as the first injection. Twelve hours after the second injection of farrerol or dexamethasone, these mice were sacrificed by cervical dislocation and the mammary glands were collected for further experiments.

### 4.3. Histopathological Examination

Mammary glands were isolated from all experimental mice, and fixed with 4% formaldehyde for 24 h. Then, the tissue blocks were dehydrated, made transparent, dipped in wax, embedded, sliced, made of the paraffin sections and then stained by means of hematoxylin–eosin (HE) staining. Finally, the histopathological changes of mammary glands were observed under a microscope.

### 4.4. Myeloperoxidase (MPO) Assay in Mammary Glands

Mammary gland samples were homogenized by adding hepesfreeacid (HEPES) and the liquid supernatant was used to perform enzyme-linked immunosorbent assay (ELISA). Cetyltrimethylammonium chloride (CTAC) was added to the precipitate, grinded and centrifuged, then collected the supernatant and used to examine the MPO by measuring the absorbance (OD) at 450 nm.

### 4.5. Enzyme-Linked Immunosorbent Assay (ELISA)

The mammary glands were homogenized after adding HEPES and the supernatants were collected by centrifugation to detect the inflammatory factors TNF-α, IL-6 and IL-1β. The levels of TNF-α, IL-6 and IL-1β in mammary glands were determined using the mouse ELISA kits (Biolegend, San Diego, CA, USA) according to the manufacturer’s instructions.

### 4.6. Cell Culture

The mMECs were purchased from the American Type Culture Collection (ATCC, ATCC^®^ CRL-3063™, Rockville, MD, USA), and these cells were cultured in DMEM medium (Gibco, Grand Island, NY 14072, USA) containing 10% FBS (Clark Bioscience, Richmond, VA, USA) at 37 °C in a humidified incubator with 5% CO_2_.

### 4.7. Cell Experimental Design

MMECs were cultured in a 60 mm × 15 mm cell culture dish (Life Science, Oneonta, NY, USA) and divided into five groups: NT group, LPS group, and LPS + farrerol (90, 110, 130 μM) groups. When 80% of the mMECs grew all over the dish, serum-containing medium in culture dish was replaced by serum-free medium. After 3 h, different concentrations of farrerol were added to the LPS + farrerol (90, 110, 130 μM) group culture dishes, respectively. Then, one hour later, an equal dose of LPS (1 μg/mL) was added to the culture dishes of the LPS group and LPS + farrerol (90, 110, 130 μM) groups. After four hours, the cells were collected.

### 4.8. Cell Counting Kit-8 Assay

The effects of farrerol on mMECs viability were determined by CCK-8 analysis. MMECs were divided into five groups: NT group and Farrerol (70, 90, 110, 130 μM) groups were added with different concentrations of farrerol. Then, five hours later, 10 μL CCK-8 (Saint-Bio, Shanghai, China) was added to each well. After one hour, the absorbance (OD) was measured at 450 nm on a microplate reader.

### 4.9. Real-Time PCR

The mRNA expression levels of TNF-α, IL-6 and IL-1β in mMECs were detected by real-time PCR. Total RNA was extracted from mMECs using TRIzol reagent in accordance with the manufacturer’s instructions. Then, 2 μg total RNA was reverse-transcribed (RT) using the PrimeScript™ RT reagent Kit (TaKaRa, Kyoto, Japan) to synthesize cDNA sequences. RT-PCR analysis of gene expression was performed using 1 μL cDNA and SYBR^®^ Green Premix Ex Taq™ II (TaKaRa, Kyoto, Japan), as recommended by the manufacturer, on the CFX96 system (Bio-Rad, Hercules, CA, USA). Synthesized cDNA was used to perform real-time PCR according to the following program: 95 °C for 30 s followed by 40 cycles of 95 °C for 5 s and 60 °C for 30 s, and a melt curve was generated from 65 °C to 95 °C (increment 0.5 °C, 5 s). Comparative quantification was performed using the 2^−ΔΔ*C*t^ method. Each treatment had three replicates.

### 4.10. Western Blot Analysis

Total proteins were isolated from mammary glands or mMECs using a radio immunoprecipitation assay (RIPA) lysis buffer (Beyotime, Shanghai, China). The supernatant was collected and the protein concentrations were determined with an Enhanced BCA Protein Assay Kit (Beyotime, Shanghai, China). Proteins were separated by SDS-PAGE and then transferred to PVDF (Millipore, Darmstadt, Germany) membrane. Then, the PVDF membrane was blocked with 5% milk in tris-buffered saline-Tween (TBST) solution for 2 h. The PVDF membrane was incubated with a primary antibody (iNOS (1:1000), COX-2 (1:1000), AKT (1:1000), phospho-AKT (1:1000), ERK1/2 (1:2000), phospho-ERK1/2 (1:2000), p38 (1:2000), phospho-p38 (1:1000), NF-κB p65 (1:2000), phospho-NF-κB p65 (1:1000) (Cell Signaling Technology, Beverly, MA, USA) and β-tubulin (1:5000) (Santa Cruz, CA, USA)) overnight at 4 °C. The PVDF membrane was washed 5 times with TBST solution for 10 min each time. Then, the PVDF membrane was incubated with secondary antibody goat anti-rabbit (1:3000) or goat anti-mouse (1:3000) (Santa Cruz, CA, USA) for 1 h at room temperature, and, with that, the PVDF membrane was washed 5 times with TBST solution for 10 min each time. The specific protein bands were obtained with the Enhanced Chemiluminescence Detection Kit (Beyotime, Shanghai, China) according to the manufacturer’s instructions.

### 4.11. Statistical Analyses

These data were analyzed using GraphPad Prism7 (Manufacturer, La Jolla, CA, USA). The differences of various experimental groups were analyzed by using one-way ANOVA combined with Tukey’s multiple comparisons test. All data are presented as means ± SEM.

## Abbreviations

mMECs	mouse mammary epithelial cells
LPS	lipopolysaccharide

## References

[1] Viguier C., Arora S., Gilmartin N., Welbeck K., O’Kennedy R. (2009). Mastitis detection: Current trends and future perspectives. Trends Biotechnol..

[2] Son S.J., Park M.R., Ryu S.D., Maburutse B.E., Oh N.S., Park J., Oh S., Kim Y. (2016). Short communication: In vivo screening platform for bacteriocins using *Caenorhabditis elegans* to control mastitis-causing pathogens. J. Dairy Sci..

[3] Ganda E.K., Bisinotto R.S., Decter D.H., Bicalho R.C. (2016). Evaluation of an On-Farm Culture System (Accumast) for Fast Identification of Milk Pathogens Associated with Clinical Mastitis in Dairy Cows. PLoS ONE.

[4] Sadek K., Saleh E., Ayoub M. (2017). Selective, reliable blood and milk bio-markers for diagnosing clinical and subclinical bovine mastitis. Trop. Anim. Health Prod..

[5] Nicholas R.A., Fox L.K., Lysnyansky I. (2016). Mycoplasma mastitis in cattle: To cull or not to cull. Vet. J..

[6] Sousaris N., Barr R.G. (2016). Sonographic Elastography of Mastitis. J. Ultrasound Med..

[7] Yang W., Zerbe H., Petzl W., Brunner R.M., Guenther J., Draing C., von Aulocke S., Schuberth H.J., Seyfert H.M. (2008). Bovine TLR2 and TLR4 properly transduce signals from *Staphylococcus aureus* and *E. coli*, but *S. aureus* fails to both activate NF-κB in mammary epithelial cells and to quickly induce TNFα and interleukin-8 (CXCL8) expression in the udder. Mol. Immunol..

[8] Bougarn S., Cunha P., Gilbert F.B., Meurens F., Rainard P. (2011). Technical note: Validation of candidate reference genes for normalization of quantitative PCR in bovine mammary epithelial cells responding to inflammatory stimuli. J. Dairy Sci..

[9] Song X., Zhang W., Wang T., Jiang H., Zhang Z., Fu Y., Yang Z., Cao Y., Zhang N. (2014). Geniposide plays an anti-inflammatory role via regulating TLR4 and downstream signaling pathways in lipopolysaccharide-induced mastitis in mice. Inflammation.

[10] Triantafilou M., Triantafilou K. (2005). The dynamics of LPS recognition: Complex orchestration of multiple receptors. J. Endotoxin Res..

[11] Van Soest F.J.S., Abbeloos E., McDougall S., Hogeveen H. (2018). Addition of meloxicam to the treatment of bovine clinical mastitis results in a net economic benefit to the dairy farmer. J. Dairy Sci..

[12] Fitzpatrick C.E., Chapinal N., Petersson-Wolfe C.S., deVries T.J., Kelton D.F., Duffield T.F., Leslie K.E. (2013). The effect of meloxicam on pain sensitivity, rumination time, and clinical signs in dairy cows with endotoxin-induced clinical mastitis. J. Dairy Sci..

[13] Zhang G.D., Wang M.Z., Zhang S.R. (1980). Studies on the quantitative determination of farrerol in Man-Shan-Hong (*Rhododendron dauricum* T.) leaves. Yao Xue Xue Bao.

[14] Liu E., Liang T., Wang X., Ban S., Han L., Li Q. (2015). Apoptosis induced by farrerol in human gastric cancer SGC-7901 cells through the mitochondrial-mediated pathway. Eur. J. Cancer Prev..

[15] Wang J., Guo C., Wei Z., He X., Kou J., Zhou E., Yang Z., Fu Y. (2016). Morin suppresses inflammatory cytokine expression by downregulation of nuclear factor-kappaB and mitogen-activated protein kinase (MAPK) signaling pathways in lipopolysaccharide-stimulated primary bovine mammary epithelial cells. J. Dairy Sci..

[16] Cao R., Fu K., Lv X., Li W., Zhang N. (2014). Protective effects of kaempferol on lipopolysaccharide-induced mastitis in mice. Inflammation.

[17] Ci X., Chu X., Wei M., Yang X., Cai Q., Deng X. (2012). Different effects of farrerol on an OVA-induced allergic asthma and LPS-induced acute lung injury. PLoS ONE.

[18] Sordillo L.M., Streicher K.L. (2002). Mammary gland immunity and mastitis susceptibility. J. Mammary Gland Biol. Neoplasia.

[19] Alluwaimi A.M. (2004). The cytokines of bovine mammary gland: Prospects for diagnosis and therapy. Res. Vet. Sci..

[20] Song X., Wang T., Zhang Z., Jiang H., Wang W., Cao Y., Zhang N. (2015). Leonurine exerts anti-inflammatory effect by regulating inflammatory signaling pathways and cytokines in LPS-induced mouse mastitis. Inflammation.

[21] De Schepper S., de Ketelaere A., Bannerman D.D., Paape M.J., Peelman L., Burvenich C. (2008). The toll-like receptor-4 (TLR-4) pathway and its possible role in the pathogenesis of *Escherichia coli* mastitis in dairy cattle. Vet. Res..

[22] Ershun Z., Yunhe F., Zhengkai W., Yongguo C., Naisheng Z., Zhengtao Y. (2014). Cepharanthine attenuates lipopolysaccharide-induced mice mastitis by suppressing the NF-κB signaling pathway. Inflammation.

[23] Wang Q., Zhang B., Yu J.L. (2016). Farrerol inhibits IL-6 and IL-8 production in LPS-stimulated human gingival fibroblasts by suppressing PI3K/AKT/NF-κB signaling pathway. Arch. Oral Biol..

[24] Saha R.N., Pahan K. (2006). Regulation of inducible nitric oxide synthase gene in glial cells. Antioxid. Redox Signal..

[25] Sil S., Ghosh T., Ghosh R., Gupta P. (2017). Nitric oxide synthase inhibitor, aminoguanidine reduces intracerebroventricular colchicine induced neurodegeneration, memory impairments and changes of systemic immune responses in rats. J. Neuroimmunol..

[26] Bortolanza M., Cavalcanti-Kiwiatkoski R., Padovan-Neto F.E., da-Silva C.A., Mitkovski M., Raisman-Vozari R., Del-Bel E. (2015). Glial activation is associated with l-DOPA induced dyskinesia and blocked by a nitric oxide synthase inhibitor in a rat model of Parkinson’s disease. Neurobiol. Dis..

[27] Yang Z., Zhou E., Wei D., Li D., Wei Z., Zhang W., Zhang X. (2014). Emodin inhibits LPS-induced inflammatory response by activating PPAR-γ in mouse mammary epithelial cells. Int. Immunopharmacol..

[28] Zhang H., Yan J., Zhuang Y., Han G. (2015). Anti-inflammatory effects of farrerol on IL-1β-stimulated human osteoarthritis chondrocytes. Eur. J. Pharmacol..

[29] Ghosh S., May M.J., Kopp E.B. (1998). NF-κB and Rel proteins: Evolutionarily conserved mediators of immune responses. Annu. Rev. Immunol..

[30] Manna S.K. (2012). Double-edged sword effect of biochanin to inhibit nuclear factor κB: Suppression of serine/threonine and tyrosine kinases. Biochem. Pharmacol..

[31] Yang Z., Yin R., Cong Y., Yang Z., Zhou E., Wei Z., Liu Z., Cao Y., Zhang N. (2014). Oxymatrine lightened the inflammatory response of LPS-induced mastitis in mice through affecting NF-κB and MAPKs signaling pathways. Inflammation.

[32] Ruifeng G., Yunhe F., Zhengkai W., Ershun Z., Yimeng L., Minjun Y., Xiaojing S., Zhengtao Y., Naisheng Z. (2014). Chlorogenic acid attenuates lipopolysaccharide-induced mice mastitis by suppressing TLR4-mediated NF-κB signaling pathway. Eur. J. Pharmacol..

[33] Li D., Zhang N., Cao Y., Zhang W., Su G., Sun Y., Liu Z., Li F., Liang D., Liu B. (2013). Emodin ameliorates lipopolysaccharide-induced mastitis in mice by inhibiting activation of NF-κB and MAPKs signal pathways. Eur. J. Pharmacol..

